# Computational aesthetics and applications

**DOI:** 10.1186/s42492-018-0006-1

**Published:** 2018-09-05

**Authors:** Yihang Bo, Jinhui Yu, Kang Zhang

**Affiliations:** 1 Department of Fine Art, Beijing Film Academy, Beijing, 100088 China; 2 The State Key Lab. of CAD&CG, Zhejiang University, Hangzhou, 310058 China; 3 Department of Computer Science, The University of Texas at Dallas, Richardson, TX 75080 USA

**Keywords:** Computational aesthetics, Aesthetic measurement, Generative art, Fractal art, Abstract painting

## Abstract

Computational aesthetics, which bridges science and art, is emerging as a new interdisciplinary field. This paper concentrates on two main aspects of computational aesthetics: aesthetic measurement and quantification, generative art, and then proposes a design generation framework. On aesthetic measurement and quantification, we review different types of features used in measurement, the currently used evaluation methods, and their applications. On generative art, we focus on both fractal art and abstract paintings modeled on well-known artists’ styles. In general, computational aesthetics exploits computational methods for aesthetic expressions. In other words, it enables computer to appraise beauty and ugliness and also automatically generate aesthetic images. Computational aesthetics has been widely applied to many areas, such as photography, fine art, Chinese hand-writing, web design, graphic design, and industrial design. We finally propose a design generation methodology, utilizing techniques from both aesthetic measurements and generative art.

## Background

The term “aesthetic” originated in Greek “aisthitiki” means perception through sensation. In Cambridge Dictionary, aesthetic is “related to the enjoyment or study of beauty”, or “an aesthetical object or a work of art is one that throws great beauty”. Aesthetics is subjective to a great extent, since there is no standard to judge beauty and ugliness. People from various domains may have totally different understandings to an art work, influenced by their backgrounds, experiences, genders or other uncertain factors.

With the rapid advances of digital technology, computers may play useful roles in aesthetic evaluation, such as aesthetic computing, making aesthetics decision, and simulating human to understand and deduce aesthetics [1]. One can use scientific approaches to measure the aesthetics of art works.

Relating to digital technology and visual art, two interdisciplinary areas emerge: *computational aesthetics* and *aesthetic computing*. Both areas focus on bridging fine art, design, computer science, cognitive science and philosophy. Specifically, computational aesthetics aims to solve the problems of how computers could generate various visual aesthetic expressions or evaluate aesthetics of various visual expressions automatically. For example, automatic generation of abstract paintings in different styles, such as those of Malevich or Kandinsky and aesthetic assessment of photo, calligraphy, painting, or other forms of art works. On the other hand, aesthetic computing aims to answer the questions of how traditional visual art theory and techniques could aid in beautifying the products of modern technology or enhance their usability. This paper will concentrate on the former, i.e., computational aesthetics.

The first quantitative aesthetic theory was proposed in “Aesthetic Measure” by Birkhoff in 1933 [2], which is considered the origin of computational aesthetics. Birkhoff proposed a simple formula for aesthetic measurement:

1 $$ M=O/C $$

where *O* is the order of the object to be measured, *C* is the complexity of the object, and *M* is the aesthetic measurement of the object. This implies that orderly and simple objects appear to be more beautiful than chaotic and/or complex objects. Often regarded as two opposite aspects, order plays a positive role in aesthetics while complexity often plays a negative role.

Birkhoff assumed that order properties, such as symmetry, contrast, rhythm, were elements, could bring comfortable and harmonious feelings. These properties apply to shape and composition at a high level, and also color and texture at a low level. Color perception is one of the most important factors for aesthetics, and color harmony [3] is frequently used to evaluate aesthetics.

On the other hand, fractal theory is another major element of aesthetics since similar objects could be perceived more easily. In 1967, Mandelbrot proposed the self-similarity of Britain coast [4], and in 1975, he created the fractal theory and studied the property and application of fractals. Spehar et al. [5] compares between fractals and human aesthetics to enable fractal theory to be a measurement of order.

According to Birkhoff, complexity is another vital factor to quantify aesthetics. People prefer simple and neat objects to complicated and burdensome ones. The more effort human visual processing system makes in viewing an object, the more complex the object is. For example, one could measure a photograph’s complexity by counting the number of objects, colors or edges in it.

**Fig. 1:**
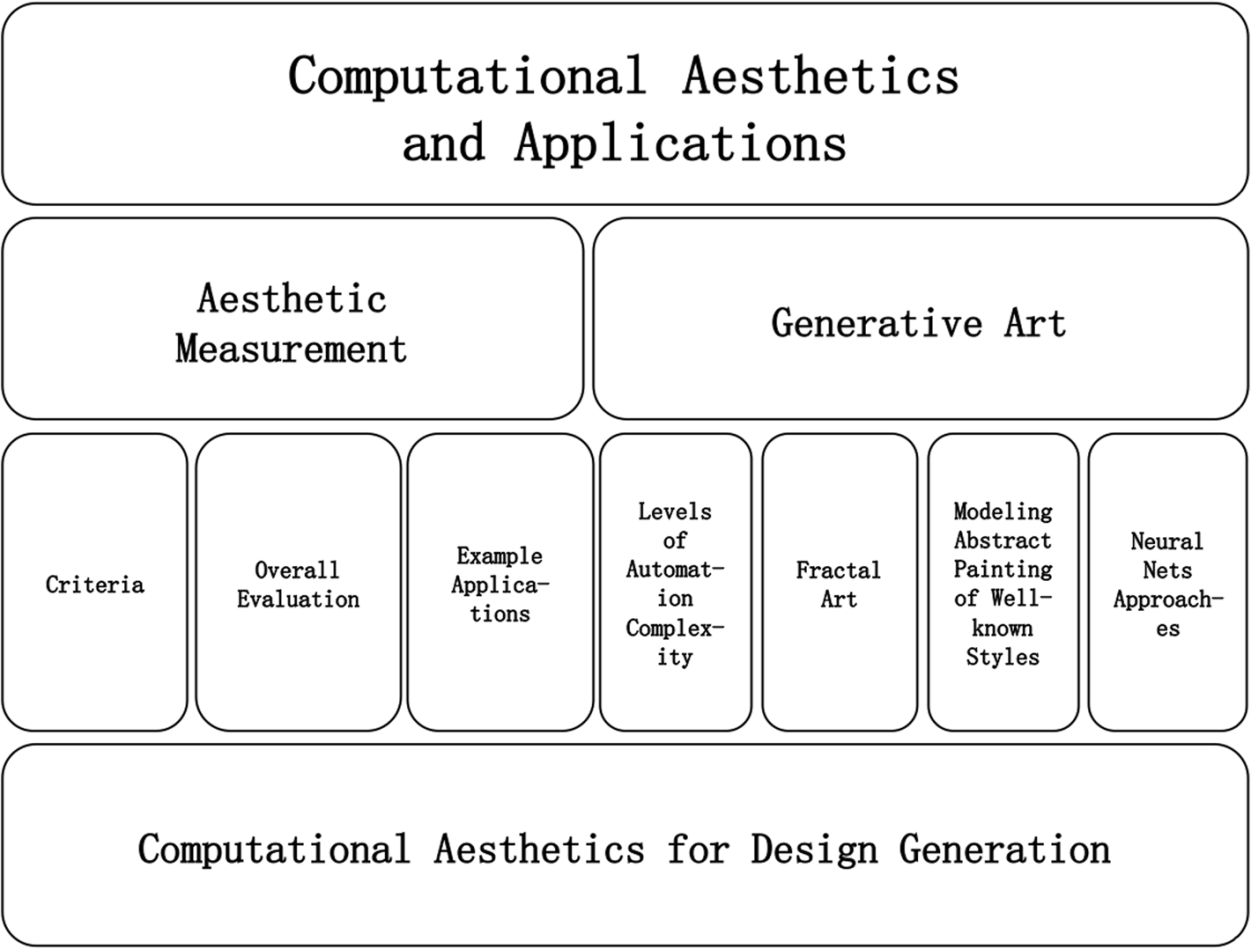
Structure of this paper

This paper concentrates on two main aspects of computational aesthetics (as shown in Fig. 1). Section “Aesthetic Measurements” describes the aesthetic criteria and measurements, and reviews their applications. Section “Generative Art” discusses generative art, including fractal art and abstract painting modeled on well-known artists’ styles. Section “Computational Aesthetics for Design Generation” proposes a design generation methodology, combining the techniques in Sections “Aesthetic Measurements and Generative Art”. Section “Conclusions” concludes the paper.

## Methods

### Aesthetic measurements

How to simulate the human visual system and brain to measure and quantify aesthetics is a great challenge. It, however, becomes possible with the rapid development of artificial intelligence, machine learning, pattern recognition, and computer vision. This section will first discuss various possible aesthetic criteria to be used for measurements, and then consider evaluation approaches using the criteria. We will then sample a few application domains.

#### Criteria

Similar to most computer vision and pattern recognition algorithms, aesthetic measurements need to consider an object’s features and their descriptions. This subsection will discuss composition criteria at a high level and image attributes at a low level.

##### Composition

Photographers always apply certain rules to make their photos appealing, including Rules of Thirds, Golden Ratio (Visual Weight Balance), focus, ISO speed rating, geometric composition and shutter speed [6]. Studies show that the photographic compositions conformed to human visual stimulation can give high aesthetic quality.

**Fig. 2:**
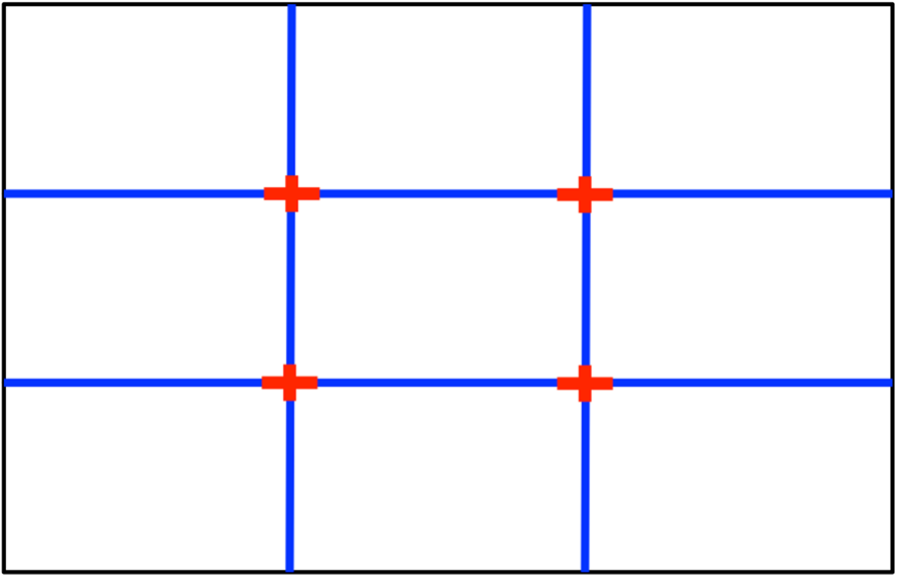
Rules of Thirds
- Bhattacharya et al. [7] define a relative foreground position to measure the coincidence of the foreground and the strong focal points. They use the following equation to characterize a 4-dimensional feature vector (F):
  2 $$ \mathrm{F}=\frac{1}{\mathrm{H}\times \mathrm{W}}\left[{\left\Vert {\mathrm{x}}_0-{\mathrm{s}}_1\right\Vert}_2,{\left\Vert {\mathrm{x}}_0-{\mathrm{s}}_2\right\Vert}_2,{\left\Vert {\mathrm{x}}_0-{\mathrm{s}}_3\right\Vert}_2,{\left\Vert {\mathrm{x}}_0-{\mathrm{s}}_4\right\Vert}_2\right] $$
- where *H* and *W* are the frame’s height and width respectively, *x*_*0*_ is the foreground object center, and *s*_*i*_ (*i* = 1,2,3,4) represents one of the four red crosses as shown in Fig. 2.
- Dhar et al. uses a similar approach to compute the minimal distance between the center of mass of the predicted saliency mask and the four red crosses [8].
- If the foreground centers at or near one of the strong focal points, the photo would become more attractive. Figure 3 shows an example with the foreground centered at two different positions in the same frame with the same background [8] In Fig. 3a, the foreground is in the middle of the frame, while in Figure 3b, the visual attention moves to the bottom-left focal point. Figure 3b appears more comfortable and harmonious than Fig. 3a. However, this measurement is only applicable to photographs with a single foreground object. Additionally, Zhou et al. [9] considers that the Rule of Thirds in saliency regions is generated by computing the average hue, saturation and value of the inner third regions, similar to the work of Datta et al. [10] and Wong et al. [11].

- **Rule of thirds** As an important guideline for photographic composition, “Rule of Thirds” means dividing a photo into 3 × 3 equal grids, as shown in Fig. 2. The four intersection points by the four dividing lines are preferred places for the photo’s main object. Placing the foreground object at an intersection point or on a dividing line would make the composition more interesting and aesthetic than placing it in the center.

**Fig. 3:**
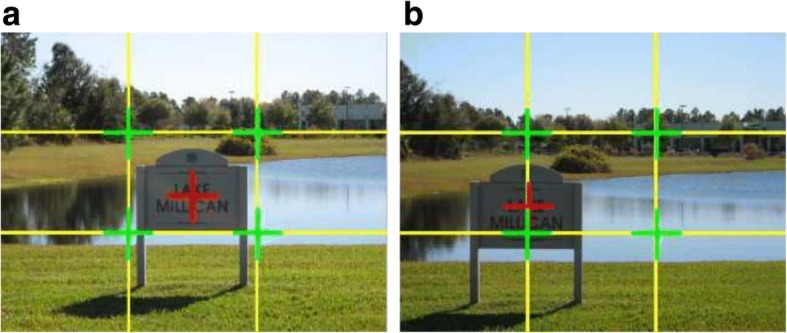
An example of foreground in the middle (**a**) and (**b**) in the one third position [3]
- **Golden Ratio** Golden Ratio, first proposed by Ancient Greek mathematicians, is also called golden mean or golden section [12]. For example, two line segments a and b are in golden ratio if (a + b)/a = a/b = (1 + √5)/2 ≈ 1.618.
- The art work “The Mona Lisa” (Fig. 4) is a perfect example of golden ratio, whether one measures the length and width of the painting or draws a rectangle around the object’s face. In photography, we often use visual weight balance or aspect ratio. Zhou et al. [9] and Datta et al. [10] consider that the image size and aspect ratio are crucial factors affecting photographs’ aesthetics. In their opinion, approximating the golden ratios, 4:3 and 16:9, can make viewers pleasing and comfortable. Obrador et al. [13] also uses photograph composition features, such as Rule of Thirds, the golden mean and the golden triangles based on edges instead of regions [14].
- **Focus, focal length and low depth of field** In photography, focus aims to adjust the distance and clarity of the frame to emphasize the foreground or salient object. Low depth of field results in the salient region or object always in sharp focus while the background is blurred. Dhar et al. [8] trains an SVM classifier on Daubechies wavelet [15] based features to calculate the blurring amount [10]. Their experiments show that low depth of field photographs receive high evaluation and rating.

**Fig. 4:**
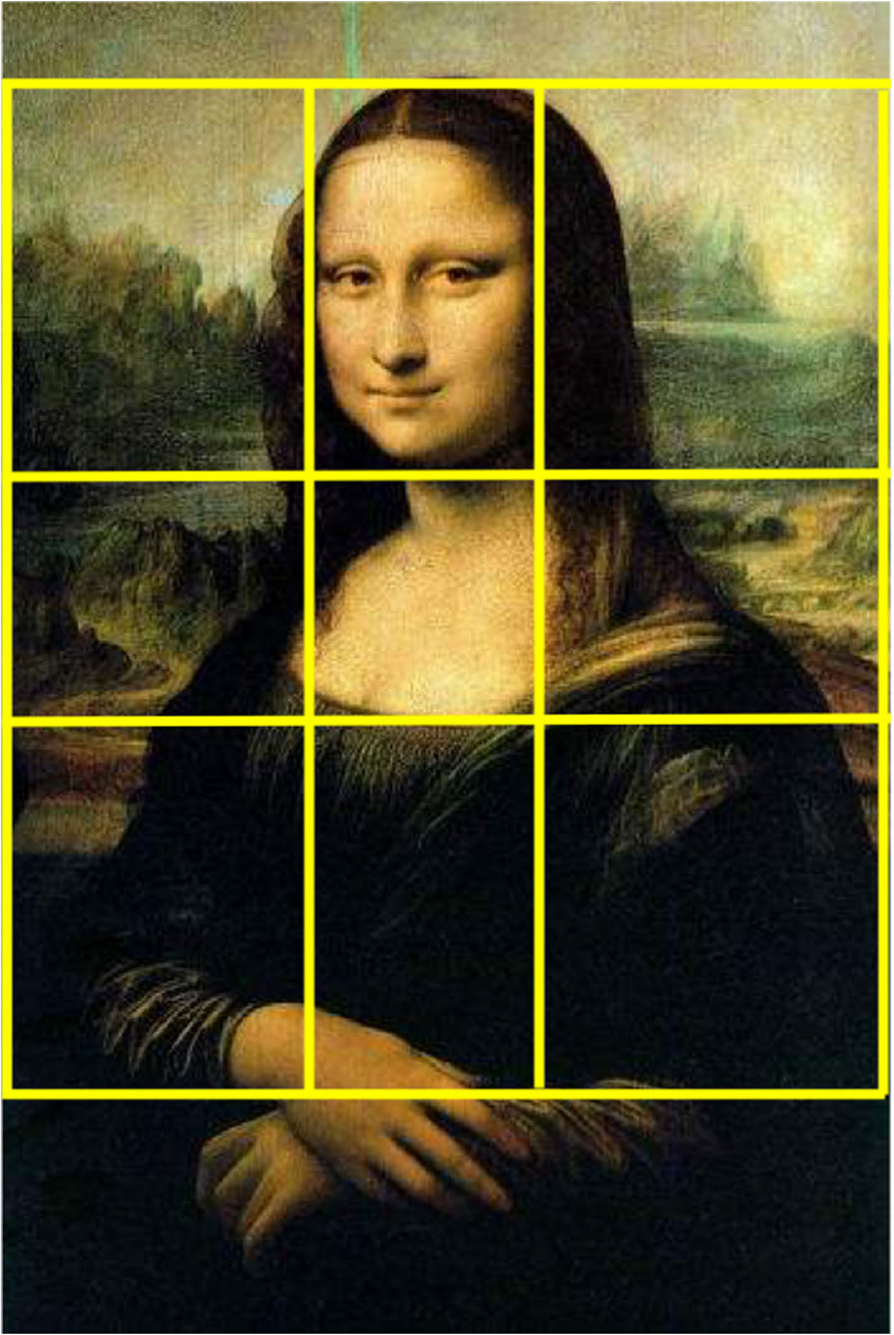
Golden Ratio example on “The Mona Lisa”

##### Imaging features

Image aesthetic features could be categorized as low-level or high-level, saliency-based, category-based, object-based, composition-based, and information theory-based. Low-level features include color, luminance, edges, and sharpness. They describe an image objectively and intuitively with relatively low time and space complexity. High level features include regions and contents.

**Fig. 5:**
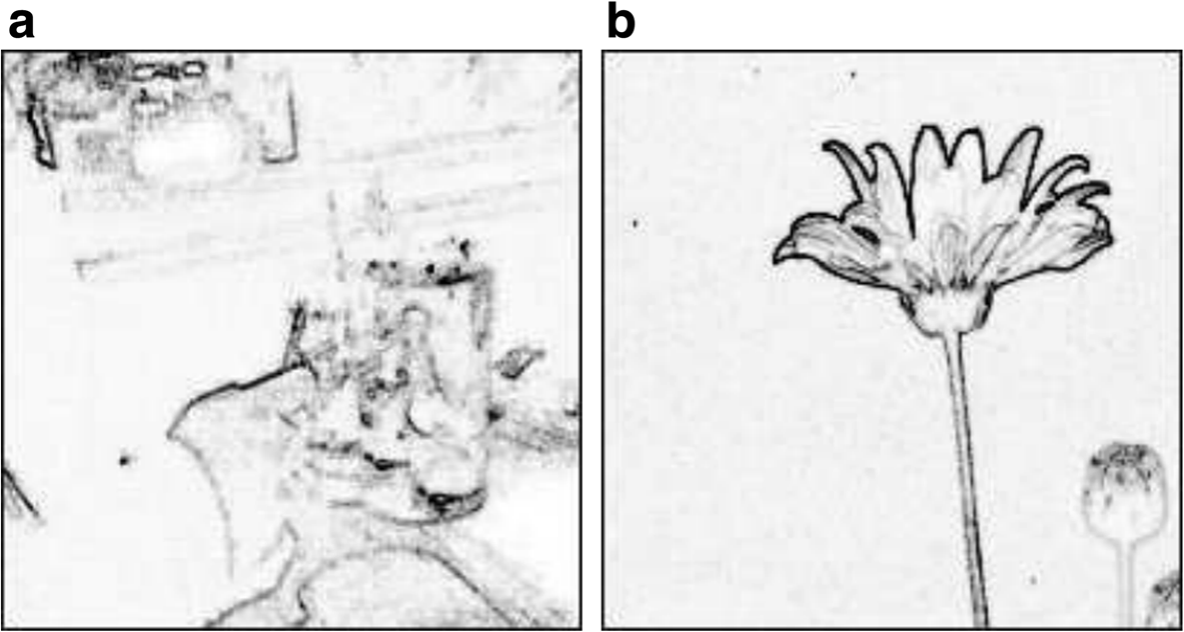
Examples of different edge distributions [30]: (**a**) an amateur photo, (**b**) a professional photo
- **Sharpness** As a feature to measure contrast, sharpness can be calculated by color, luminance, focus or edges. Obrador et al. [18] measures sharpness by contrasting edges. High contrast edges usually generate high sharpness. Wong et al. [11] extends Fourier transform [30] to compute sharpness.
- **Regions** Apart from the low-level features mentioned above, regions and contents may be considered high level features. An appealing image does not mean all of its regions to be aesthetic. Hence, researchers attempt to estimate regions’ rather than the entire image’s aesthetics. Usually, regions are segmented before aesthetic assessment.
- Obrador et al. [18] develop a region-based image evaluation framework, which includes measurements of sharpness, contrast and colorfulness. All of these region features are composed to render an appealing map. Exposure, size and homogeneity measurements of the appealing map are then applied. Zhou et al. [9] propose to use salient region detection for photograph aesthetic measurement. Wong et al. [11] use Itti’s visual saliency model [31] to obtain the salient locations of a photograph, and then compute the exposure, sharpness and texture details of these salient regions. Their approach also analyzes the position, distribution and the number of salient locations to obtain other evaluation results. Similarly, Obrador et al. [13] generates contrast regions, rather than salient regions, by analyzing five low-level features, including sharpness or focus, luminance, chroma, relevance and saliency. The approach generates five segmentation maps to aid aesthetic measurement.
- **Contents** The contents of a photograph always make great contributions to human aesthetic judgment. Different types of objects would give viewers different visual experiences. People are the most common target in photography. Dhar et al. [8] estimate whether there are people in a photograph by a face detection method [32]. If the detected face area is larger than 25% of the whole image, it is considered a portrait depiction. Meanwhile, the presence of faces [18, 20, 33] is another key factor that impacts the photograph’s appeal. Based on the face detection result, one may calculate the aesthetic score by assessing the size, color, and expression of the face [34]. Except the average luminance, contrast, color and size of the face, Obrador et al. [18] detect smile [35] with a probability, if the face region is over 3.6%.
- Another approach trains a SVM classifier to judge the presence of animals in photographs [8]. It also divides the content into indoor and outdoor scenes, and proposes 15 attributes to describe various general scene types. For outdoor scenes, it uses sky-illumination attributes to measure the lighting, which effect the perception of photographs. Photos taken in a sunny day give a clear sky, while those taken in a cloudy day give a dark sky. Obviously, photos with a clear sky appear aesthetic.

- **Color** Color is one of the most important low-level features [16, 17]. In computational aesthetics, we usually measure color in terms of colorfulness, color harmony, and apposing colors.
- *Colorfulness* [10, 13, 18–21] is decided by average Chroma and the spread of Chroma distribution, computed by brightness and saturation in a 1D form. Specifically, average Chroma presents the average distance of color to neutral axis.
- Hasler and Suesstrunk [17] propose an approach for measuring colorfulness, via an image’s color pixel distribution in the *CIELab* color space. Colorfulness (CFN) is a linear combination of color variance and Chroma magnitude:
  3 $$ \mathrm{CFN}={\sigma}_{ab}+0.37\bullet {\mu}_{ab} $$
- where ${\upsigma}_{\mathrm{a}\mathrm{b}}=\sqrt{\upsigma_{\mathrm{a}}^2+{\upsigma}_{\mathrm{b}}^2}$, represents the trigonometric length of the standard deviation in *ab* space, and *μ*_*ab*_ represents the distance of the center of gravity in the *ab* space to the neutral axis.
- The experiment by Obrador et al. [18] shows that a colorful image could receive a high rating in image appeal even though the image’s content is not attractive at all.
- As another important factor for image quality, *color harmony* is a color combination harmonious to human eyes and sensation. In general, color harmony studies which colors are suitable for simultaneous occurrence. This theory is based on the color wheel, on which the purity and saturation increase along the radius from the center outward. In other words, the color in the center of the circle has the lowest purity and saturation.
  Lu et al. [22] divide current color harmony models into two types: empirical-based [23–25] and learning-based [26–29]. The former, defined by designers or artists, appears to be subjective, while the latter behaves rationally and objectively. Most of the learning models focus on tuning the parameters on training the sample data. To make the two distinct models benefit each other, Lu et al. [22] proposes a Bayesian framework to build color harmony. Photos with harmonious colors appear comfortable to human and are usually rated with high aesthetic scores.
- *Opponent color* theory, on the other hand, states that human eyes could perceive light in three opposing components, i.e., light vs. dark, red vs. green, and blue vs. yellow. One could not sense the mixtures of red and green, or blue and yellow. Therefore, in reality, there is no such a color perceivable by human that is reddish green, or bluish yellow. Opposing colors can create maximum contrast and stability of an image. Photos or images with opposing colors are more appealing than those without. In addition, complementary colors occurring simultaneously in a photo can also enhance the foreground saliency.
- Dhar et al. [8] trains a classifier to calculate and predict complementary colors, useful for aesthetic assessment. Ke et al. [30] uses color contrast as one of their aesthetic assessment criteria. They believe that foreground and background should have complementary colors to highlight prominent subjects.
- **Luminance and exposure** In addition to color, illuminance and exposure are two other important factors for photograph aesthetic assessment. Researchers use overexposure and underexposure to penalize the overall image appeal by calculating the luminance distribution. For example, Obrador and Moroney [18] use average luminance histogram and standard deviation to measure penalization. The more luminance values, the less penalty is imposed. Wong and Low [11] consider that a professional photograph should be well exposed. Obrador et al. [13] uses luminance to compute the contrast of a region.
- **Edges** Researchers have also proposed to use edge spatial distribution [11, 30] to measure the simplicity of photos and images. A simple photo should have a salient foreground and concise background. Figure 5a gives an example by an amateur photographer with noisy background and obscure foreground. The edge spatial distribution appears rambling. On the other hand, Fig. 5b shows an edge map of a professional photograph. The foreground contour stands out clearly with few edges in the background. Ke et al. [30] proposes two different methods to measure the compactness of the spatial distribution of edges. A compact and clear distribution of edges in a photograph makes the photograph visually aesthetic.

#### Overall evaluation

After selecting and measuring appropriate features, combining their aesthetic scores to make overall evaluation is the next step. There are two types of evaluation methods: binary classification and rating. A binary method classifies photos into beautiful and unbeautiful. A rating method scores photos according to their appeal, typically from 1 to 10.

##### User studies

Obrador et al. [18] conduct user surveys to help selecting appropriate features. They give image appeal six ratings: excellent, very good, good, fair, poor and very poor. For example, excellent photos require higher sharpness, while very good ones need not be in perfect focus. The results of user surveys could also provide reliable basis for feature selection. Users are asked to list and sort the features used in their aesthetic measurement. The selected features can be used for automatic aesthetic quantification.

##### Classifiers

Support Vector Machine (SVM) is one of the most popular methods for binary classification [8]. Based on aesthetic features, one may train SVM classifiers on a labeled training data and classify photos into professional and amateur, or appealing and unappealing. Zhou et al. [9] choose the *liv*-SVM algorithm, and use the standard RBF kernel to perform classification. The n-fold cross-validation runs 10 times per feature by and filters out top 27 features. Then a greedy algorithm is used to find the top 15 features among the 27 to build a SVM classifier. Although SVM is a strong binary classifier, it performs poorly when many irrelevant features exist. CART (Classification and Regression Tree) [36] is a tree-based and fast approach, which can help analyzing the influence of various features.

Probabilistic methods [30, 37] are also commonly used for classification. Given a set of aesthetic quality metrics, researchers usually create a weighted linear combination of metrics. Ke et al. [30] propose a Naïve Bayes classifier using the following equation:

4 $$ \mathrm{q}=\frac{P\left( pr|{q}_1,{q}_2,\dots \dots, {q}_n\right)}{P\left( am|{q}_1,{q}_2,\dots \dots, {q}_n\right)}=\frac{P\left({q}_1,{q}_2,\dots \dots, {q}_n| pr\right)\mathrm{P}(pr)}{P\left({q}_1,{q}_2,\dots \dots, {q}_n| am\right)P(am)} $$

However, one cannot ensure the independence of the features. If any features are interrelated, they would be inoperative.

#### Example applications

Apart from photographs and images as discussed above, computational aesthetics has been applied to other fields, such as paintings, handwritings, and webpages, as summarized below.

##### Paintings

The aesthetics of digital or digitized paintings is subjective, varied due to different painters, types of paintings, drawing techniques, etc.. Li et al. [38, 39] build an aesthetic visual quality assessment model, which includes two steps. Step one is a questionnaire. Participants are asked to list at least two factors that affect the aesthetics of paintings. These factors are then grouped into color, composition, content, texture/brushstroke, shape, feeling of motion, balance, style, mood, originality, and unity. Step two is a rating survey. The assessment model uses the survey data to perform training and testing.

One could consider aesthetic visual assessment of paintings a machine learning problem. Using the prior survey results and knowledge, one could generate features to represent the given image both globally and locally. Global features include color distribution, brightness, blurring, and edge distribution. Local features include segment shape and color, contrast between segments, and focus regions. Given the global and local features, one could use Naïve Bayes classifier to classify paintings into high-quality and low-quality categories. Assumed independent from each other, the features are given equal weights.

**Fig. 6:**
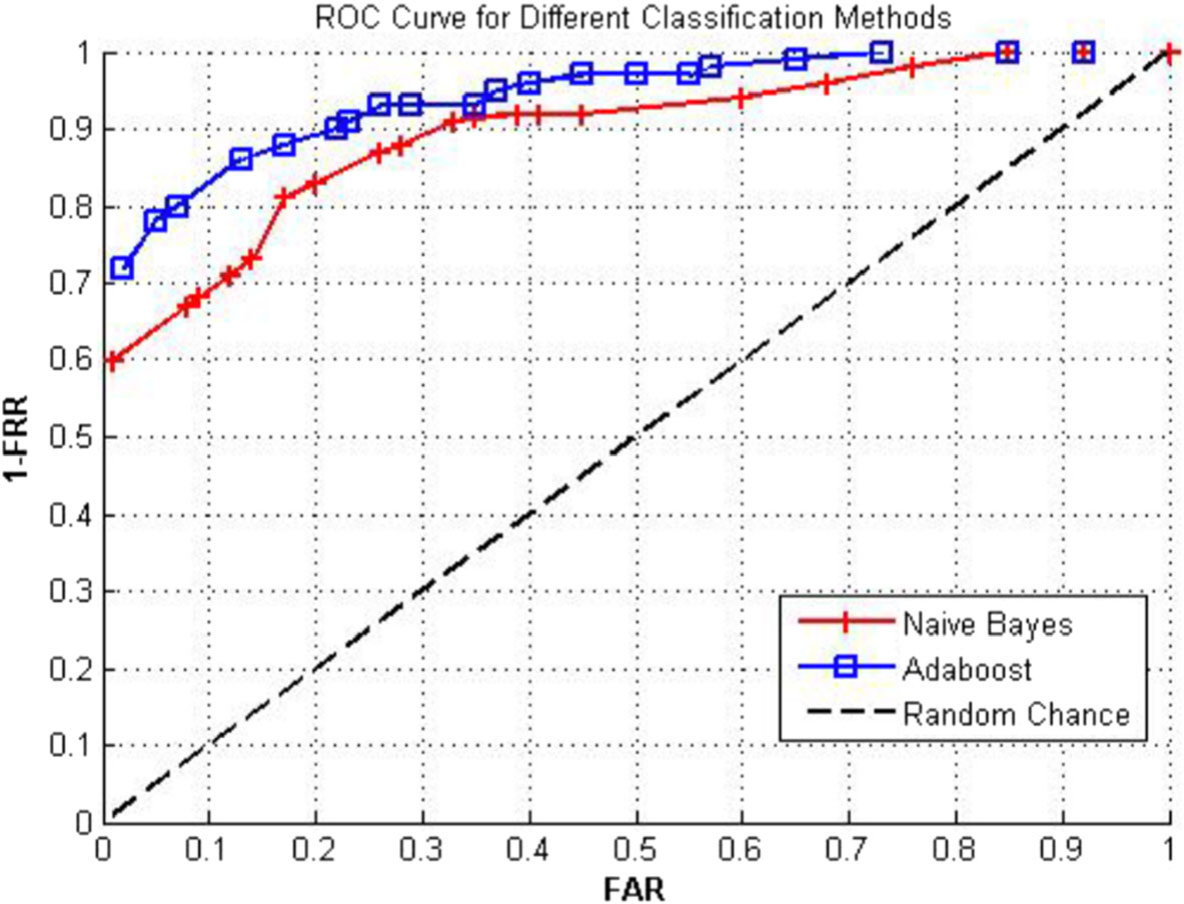
Comparison of Naïve Bayes and AdaBoost methods [0]

Different types of features may, however, carry unequal weights in an aesthetic assessment. AdaBoost [40] assigns different weights adaptively. It performs better than Naïve Bayes classifier, but both perform distinctly better than the random chance approach, as shown in Fig. 6.

**Fig. 7:**
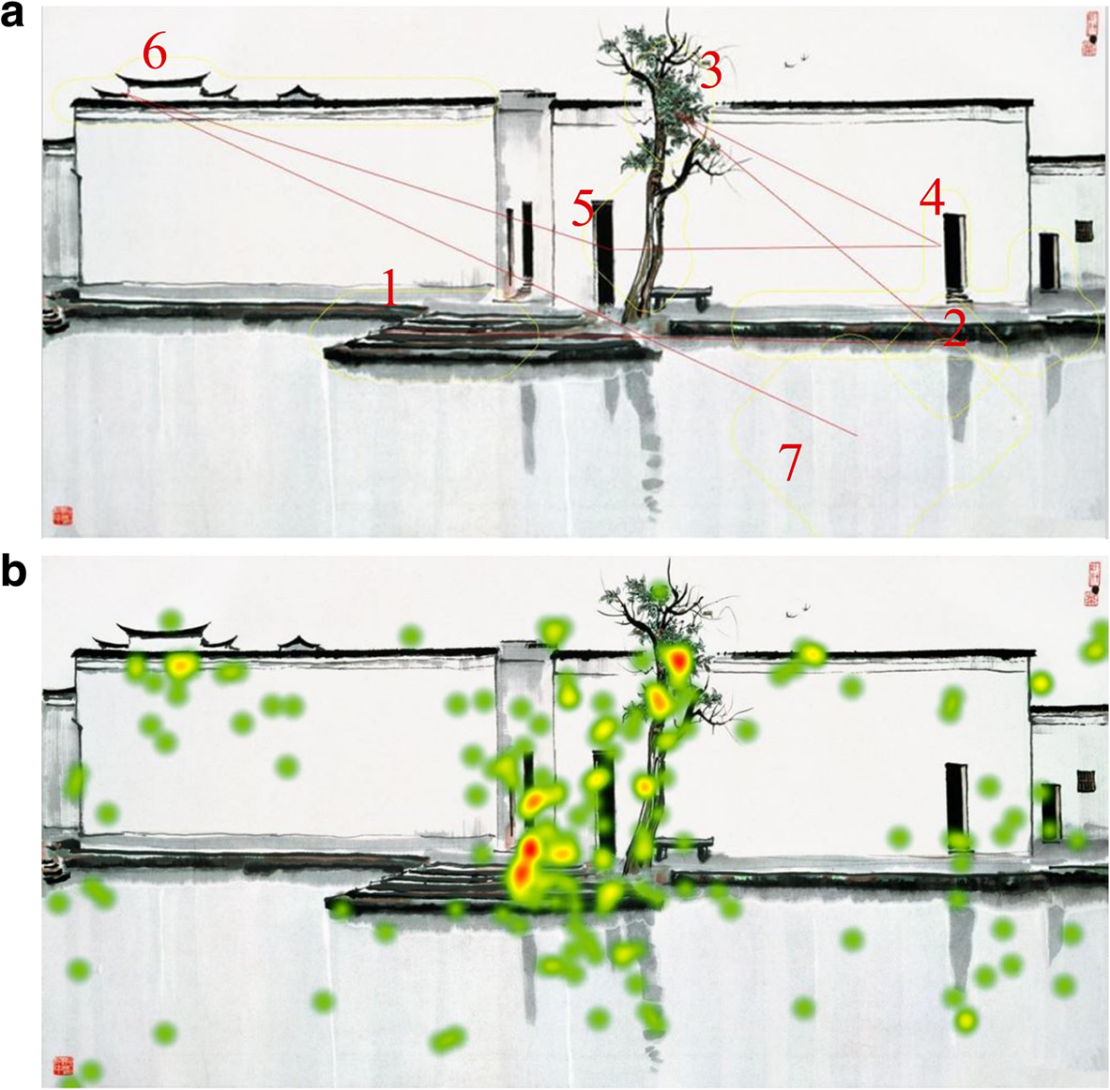
Calculated saliency result of “Twin Swallows” (**a**) and heat map of eye movements on Wu Guanzhong’s “Twin Swallows” (**b**)

Aiming to discover whether white space in Chinese ink paintings is not simply a blank background space but rather meaningful for aesthetic perception, Fan et al. [41] examines the effect of white space on perceiving Chinese paintings. Applying a computational saliency model to analyze the influence of white space on viewers’ visual information processing, the authors conducted an eye-tracking experiment. Taking paintings of a well-known artist Wu Guanzhong in a case study, they collect users’ subjective aesthetic ratings. Their results (Fig. 7) show that white space is not just a silent background: it is intentionally designed to convey certain information and has a significant effect on viewers’ aesthetic experience.

**Fig. 8:**
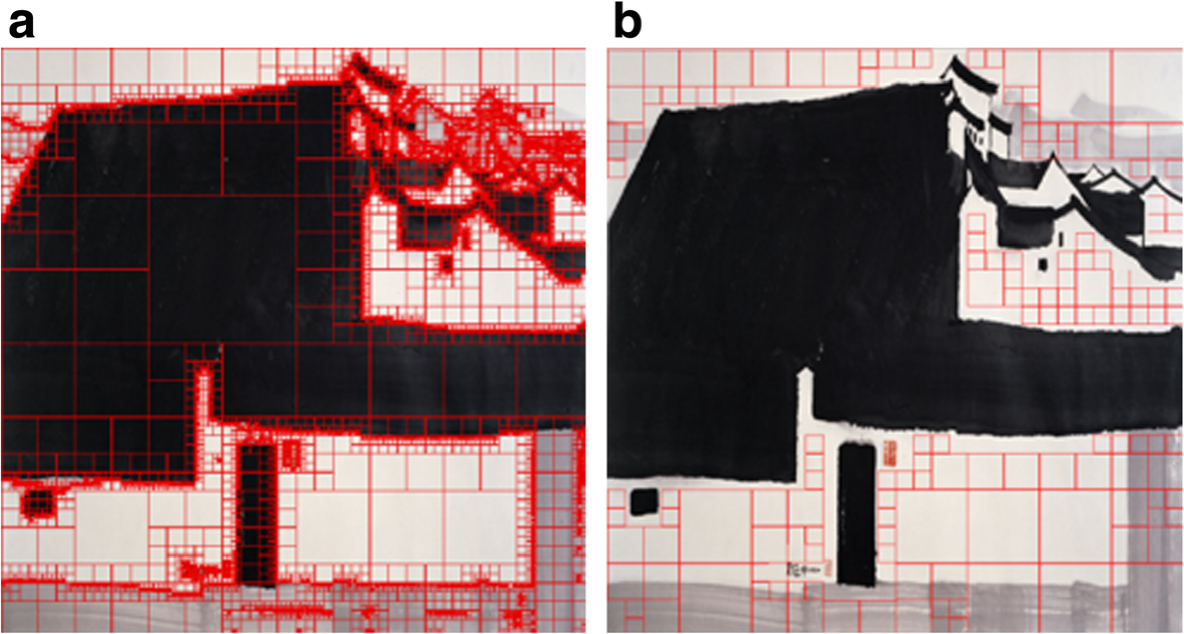
**a** An example of a quadtree decomposition on “A Big Manor”. **b** The quadtree decomposition result of white space in “A Big Manor”

Fan et al. [42] further quantifies white space using a quadtree decomposition approach, as shown in Fig. 8, in computing the visual complexity of Chinese ink paintings. By conducting regression analysis, they validate the influences of white space, stroke thickness, and color richness on perceived complexity. Their findings indicate that all above three factors influence the complexity of abstract paintings. In contrast, mere white space influences the complexity of representational paintings.

##### Chinese handwriting

Due to the special structure of Chinese handwritings, one should design features different from those for photos or paintings. Sun et al. [43] propose two types of features based on the balance between feature generality and sophistication, i.e., global features and component layout features.

**Fig. 9:**
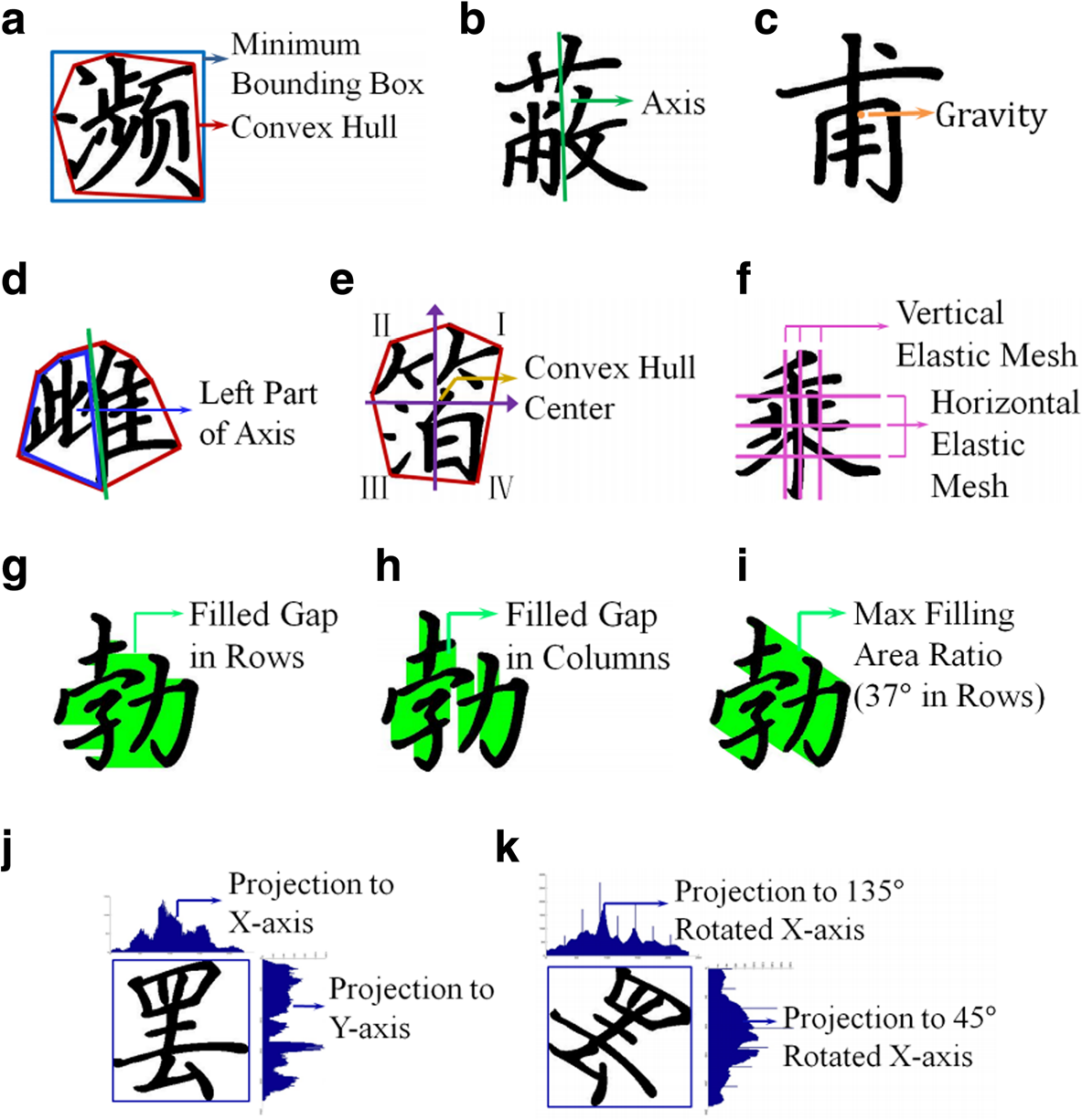
Global features of Chinese handwritings [43]
- Write Space Ratio (WSR) is a common aesthetic rule in calligraphy, representing the crowdness of characters. Sun et al. [43] use convexity, ratio of axis cutting convex hull, ratio of pixel distribution in quadrants and elastic mesh layout to evaluate the distribution of white space.
- The orientation and position of a character stroke also influence the aesthetics of handwriting. With Chinese characters, there are four types of strokes projected to 0^o^, 45^o^, 90^o^ and 135^o^ rotated X-axis respectively. Sun et al. [43] use the variance of each pixel’s projection and maximum gap proportion to measure the gap between strokes.
- **Component layout features** Apart from the above global features, layout features divide every Chinese character into several components, each constructed by a set of strokes. As shown in Fig. 10, a component feature vector is constructed by the horizontal overlap, vertical overlap, area overlap and distance between points from two different components.

- **Global features** Global features refer to three aesthetic aspects: alignment and stability, distribution of white space, gap between strokes. As shown in Fig. 9, Sun et al. [43] use the rectangularity of convex hull, slope and intersection of axis, and center of gravity to measure alignment and stability. A larger rectangularity value of the convex hull represents more regular and stable handwriting. Slope and intersection of axis divides a character into two subsets. A symmetrical and balanced character should have an approximately perpendicular axis. The center of gravity is inside the bounding-box of the character, which could describe the stability of handwriting from the perspective of physics.

**Fig. 10:**
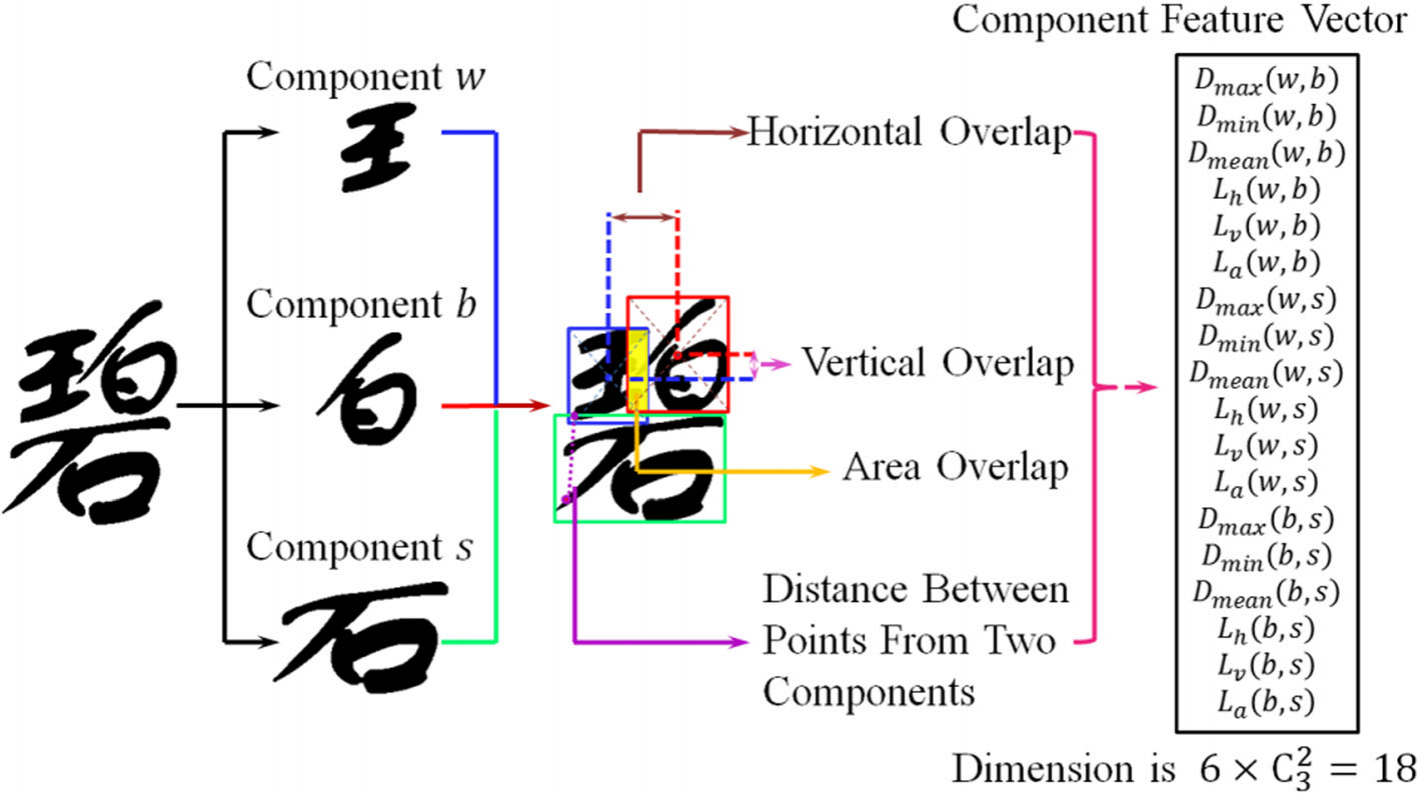
Examples of component layout [43]
- Similar to paintings, no public Chinese handwriting datasets are available for aesthetic evaluation. Sun et al. [43] build a dataset for this purpose based on the agreement in aesthetic judgments of various people. They compute the Chinese Character aesthetic score by
  $$ \mathrm{S}=100\times {\mathrm{p}}_{\mathrm{g}}+50\times {\mathrm{p}}_{\mathrm{m}}+0\times {\mathrm{p}}_{\mathrm{b}} $$
- which could give an average human evaluation score. Variables p_g_, p_m_, and p_b_ are probabilities for good, medium and bad respectively.
- To evaluate the aforementioned features, the authors construct a back propagation neural network, and show that their approach gives a comparable performance with human ratings.

##### Web pages

Researchers have also attempted to study the relationships between webpages’ computational aesthetic analysis and users’ aesthetic judgment [44]. Thirty web pages are selected from different types of network sources with various visual effects. The participants include six women and 16 men with normal vision and non-color blindness, who are tested independently. Each participant labels a page component on a 7-point scale for repelling to appealing, complicated to simple, unprofessional to professional, and dull to captivating.

**Fig. 11:**
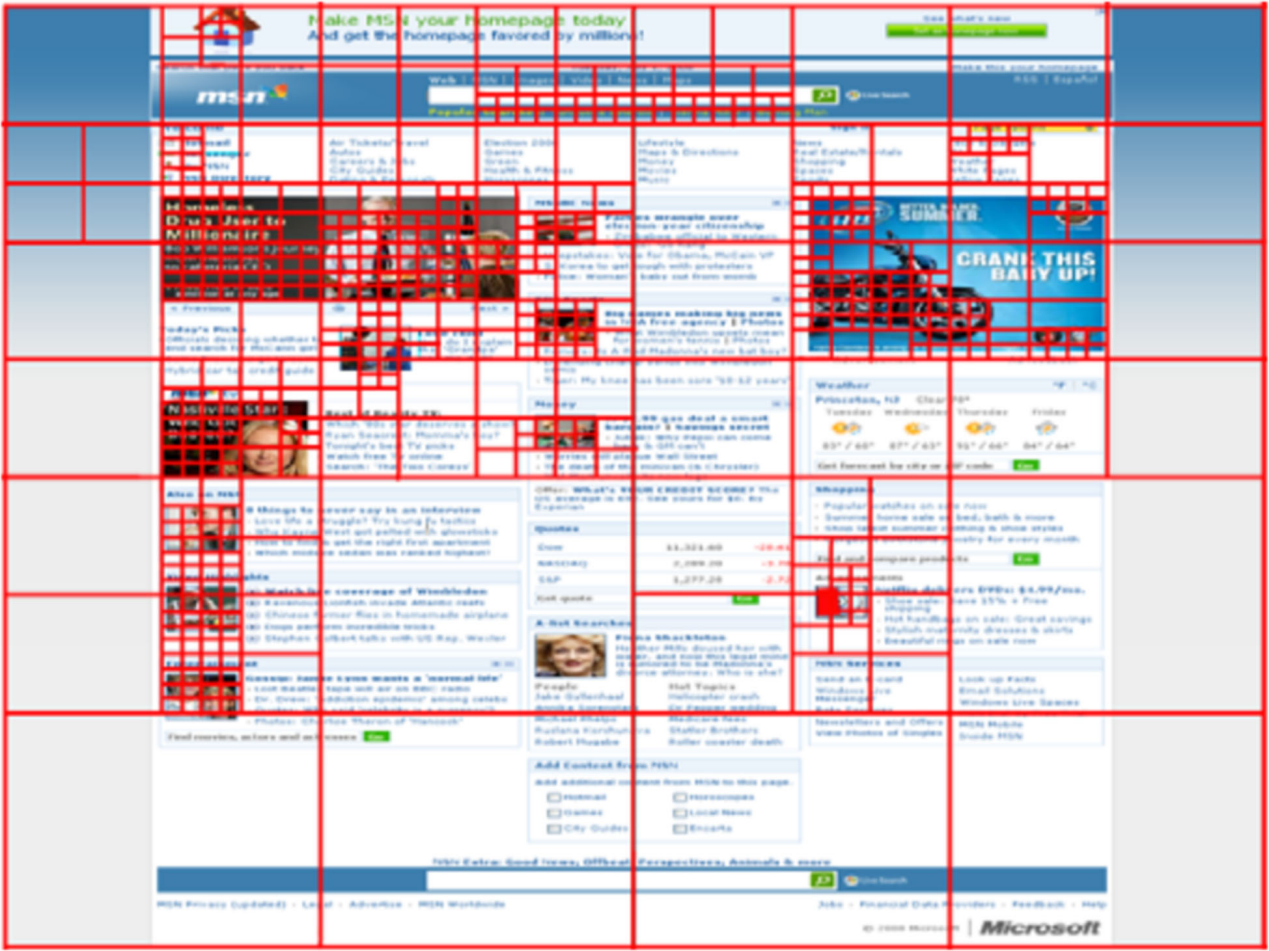
Quad-tree decomposition of a web page [44]

For computational aesthetic analysis, Zheng et al. [44] compute the aesthetics based on low-level image statistics including color, intensity and texture, regions of minimum entropy decomposition. They also evaluate the quad-tree on aesthetic dimensions, including symmetry, balance, and equilibrium, as shown in Fig. 11. They find that the human subjective ratings and computational analysis on several aforementioned aspects are highly correlated.

##### Logo designs

**Fig. 12:**
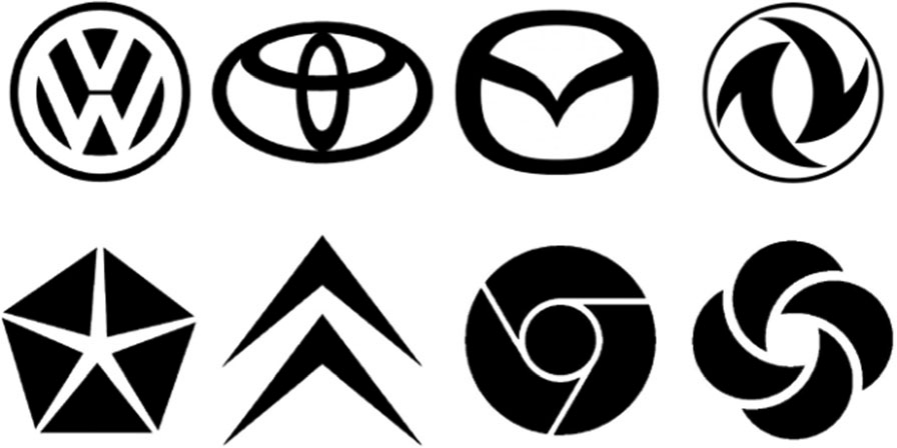
Examples of black and white logos [45]

The last application example is the evaluation of logos [45, 46], exampled in Fig. 12. The authors select features, such as balance, contrast and harmony based on design principles. To obtain reliable training data, they also collect human ratings of the above features. Using a supervised machine-learning method to train a statistical linear regression model to perform the evaluation, they are able to obtain a high correlation of 0.85.

## Generative art

Digital art becomes increasingly expressive and humanized. With the emergence and development of computational aesthetics, advanced artificial intelligence technology could help to generate interesting and unique art works.

### Levels of automation complexity

Machine intelligence is the key to computer-generated abstract paintings. We may classify computer-generated abstract paintings into four levels based on their computational complexity rather than their visual complexity [47].

Level 1 needs full human participation using an existing painting software or platform. First, software producer prepares various visual components either generated manually or automatically. Users can select visual components or draw them using the digital brush provided by the software. Of course, they can change visual attributes as needed.

The best representative of Level 2 is fractal art, originated in late 1980s [48]. Fractals require users to provide various attributes, styles and mathematical formulas as inputs. Then a programmed computer can generate results automatically. In other words, at Level 2, results are usually generated based on mathematical formulas parameterized with certain degrees of randomness. The next section will discuss fractal art further.

Methods at Level 3 are often heuristics-based using knowledge-based machine intelligence. There are two general approaches in producing abstract paintings at this level: generative and transformational. Using the generative method, one encodes artists’ styles into computational rules or algorithms. One of the pioneering works by Noll [49] makes a subjective comparison of Mondrian’s “Composition with Lines” with computer-generated images. On the other hand, a transformational method attempts to transform digital images into abstract paintings using image processing techniques. For example, a transformational method can mimic brush strokes or textures and apply them on an input image to transform it into an abstract picture [50]. The best representative of transformational methods is the so-called non-photorealistic rendering [51], which is out of the scope for this paper.

Level 4 is an AI-powered and promising direction for approaches in generating highly creative artistic and design forms. For instance, such an approach detects specific styles from existing paintings and give an objective aesthetic evaluation automatically, or the results will be adaptive to audiences’ emotional and cultural background. The current advances in deep learning and artificial intelligence have created tremendous opportunities for breakthroughs at this level.

### Fractal art

Fractal geometry, coined by mathematician Benoit Mandelbrot (1924–2010) in 1975, studies the properties and behavior of fractals and describes many situations that cannot be explained easily by classical geometry. Fractals have been applied to computer-generated art and used to model weather, plants, fluid flow, planetary orbits, music, etc. Different from traditional art, fractal art realizes the unity of math and art aesthetics. A curve is the simplest and classical expression in fractal art, which can be generated recursively or iteratively by a computer program.

We can easily discover four characteristics of fractal art works:

- Self-similar: enlarge the local part of a geometry object, if the local part is similar to the entire object, we call it self-similar.
- Infinitely fine: It has fine structure at any small scale.
- Irregularity: one cannot describe many fractal objects using simple geometric figures.
- Fractional dimension: generated based on fractal theory, fractional dimension is an index for characterizing fractal patterns or sets by quantifying their complexity.

**Fig. 13:**
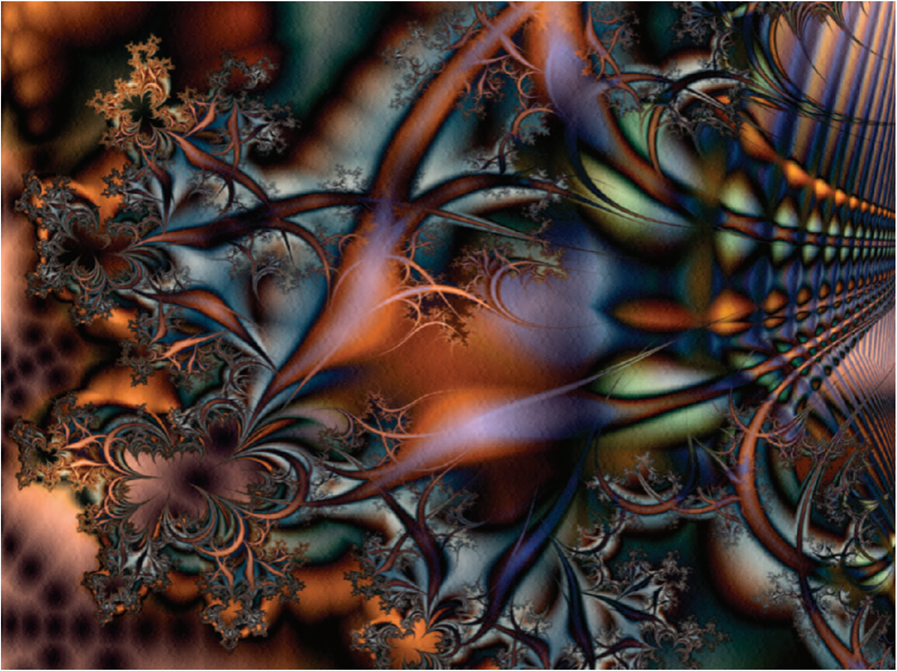
An example of combined result [52]

Singh [52] believes that there is a conversation between him and his computer when he creates his images. In other words, when he talks to his computer, the computer functions would be the translator. He builds on elements library and uses types of string fractals as compositional elements but not the main subject in the image. Figure 13 shows an example of combined result used in the Unfractal series.

**Fig. 14:**
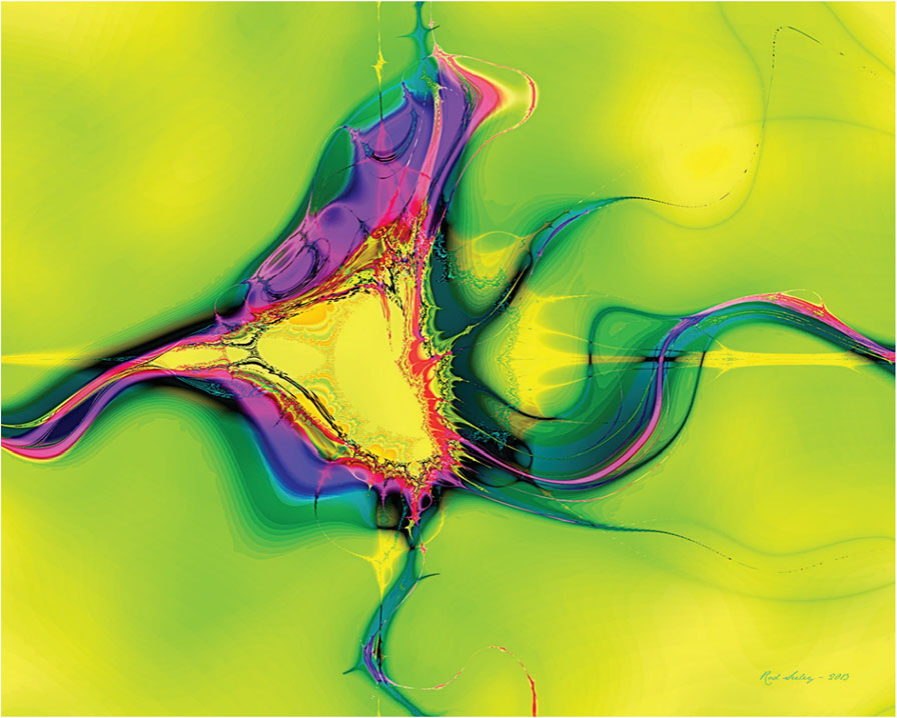
*Yellow Dreamer* [53]

Seeley uses fractals as the beginning of his art works [53], making them look less like computer-generated. A number of fractal software may be used to create this type of artworks, such as Fractal Studio, Fractal Explorer, Apophysis, ChaosPro, and XaoS. As shown in Fig. 14, named *Yellow Dreamer,* Sheeley creates the base image using Fractal Studio, and then transforms it with Filter Forge filters, Topaz Adjust 5, and AKVIS Enhancer.

### Modeling abstract painting of well-known styles

According to Arnheim [54], abstract art uses a visual language of shape, form, color and line to create a composition which may exist with a degree of independence from visual references in the world. It is thus clear that a large variety of styles of abstract paintings exist. Accordingly, style analysis is an essential step in generative art, which involves analyzing basic components, background color, component colors and their layout. The components may be independent from each other or dependent with certain rules among them. Geometrical components could be easily modeled by computers while interweaved irregular shapes could be modeled using a layered approach.

#### Style analysis

Abstract paintings may be divided into two classes, i.e. geometric abstraction and lyrical abstraction. Here we begin with the pioneer of abstract paintings, Wassily Kandisky, to analyze the style of his abstract paintings during his Bauhaus years (1922–1933), such as “composition VIII” (1923), “black circles” (1924), “Yellow Red Blue” (1925), “several circles” (1926), “Hard But Soft” (1927) and “thirteen rectangles” (1930).

**Fig. 15:**
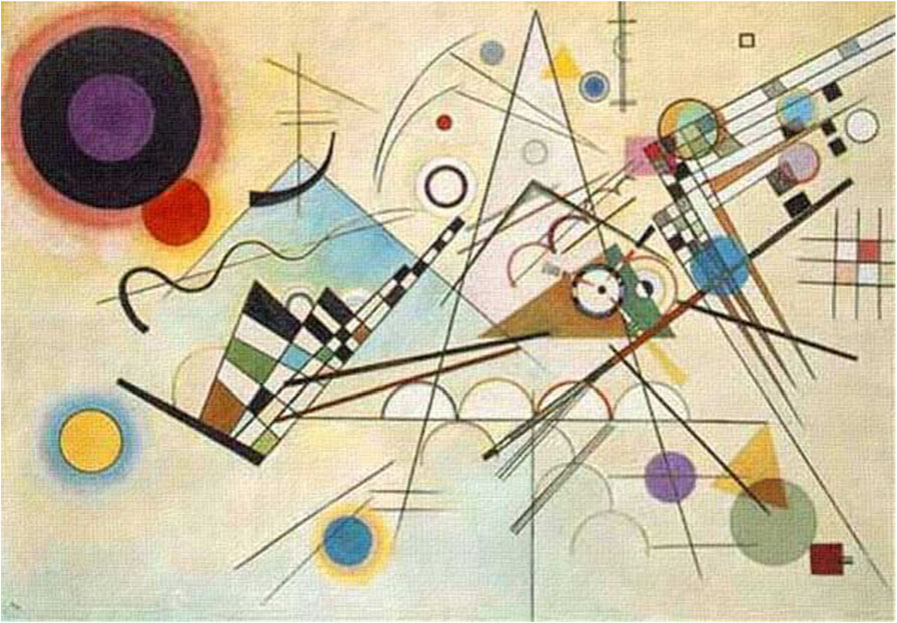
Kandinsky’s “Composition VIII”

We take “Composition VIII” shown in Fig. 15 as an example. According to Kandinsky himself, three primary forms occur frequently: sharp angles in yellow, circles in deeper colors, lines and curves in yellow and deeper colors respectively. He also proposed three pairs of contrast forms:

1. The contrast color pair: yellow vs. blue. For example, a yellow circle is always nested inside a blue circle, or vice versa.
2. A straight line is intersected with a curve line. Several straight lines are intersected with a curve line or individual lines and curves. Some lines are in one color, while others are in segmented colors.
3. Circle(s) with triangle(s). One circle overlaps one triangle, multiple circles overlap one triangle, or several abreast half circles.

**Fig. 16:**
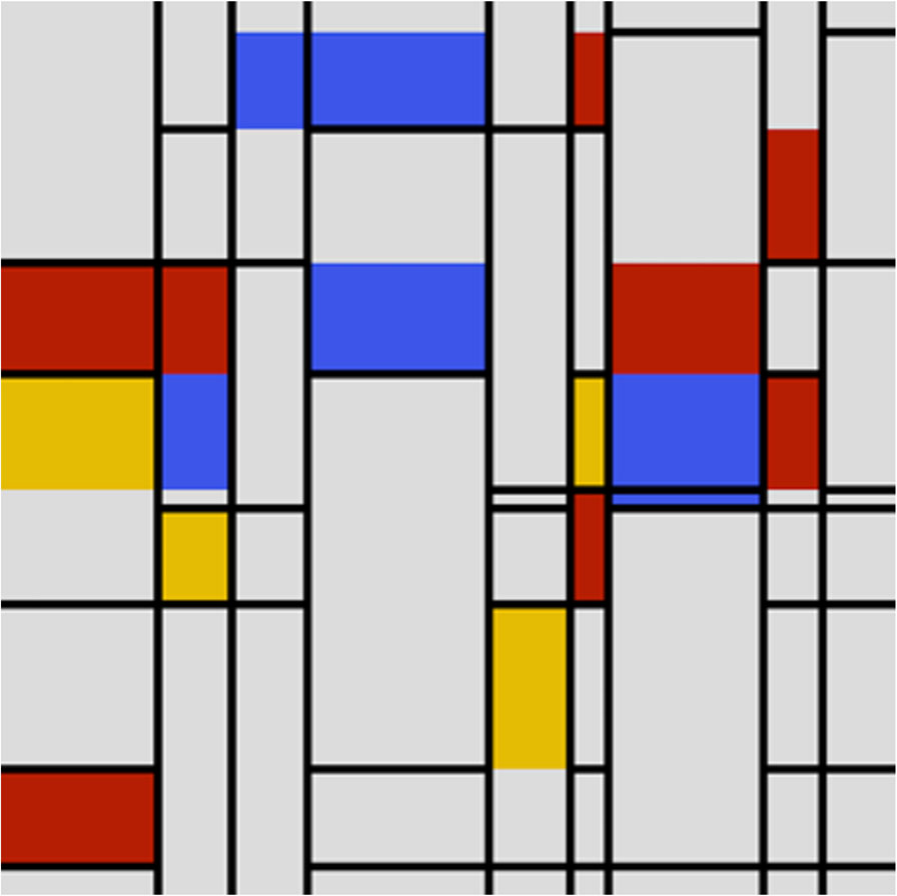
Computer generated Mondrian’s abstract painting example

Piet Mondrian is another well-known abstract artist, whose style is based on geometric and figurative shapes. While his art forms are drastically different from Kandinsky’s, he took black vertical and horizontal lines as the principal elements and used primary colors red, yellow, blue to fill some of the grids, as modeled in Fig. 16.

**Fig. 17:**
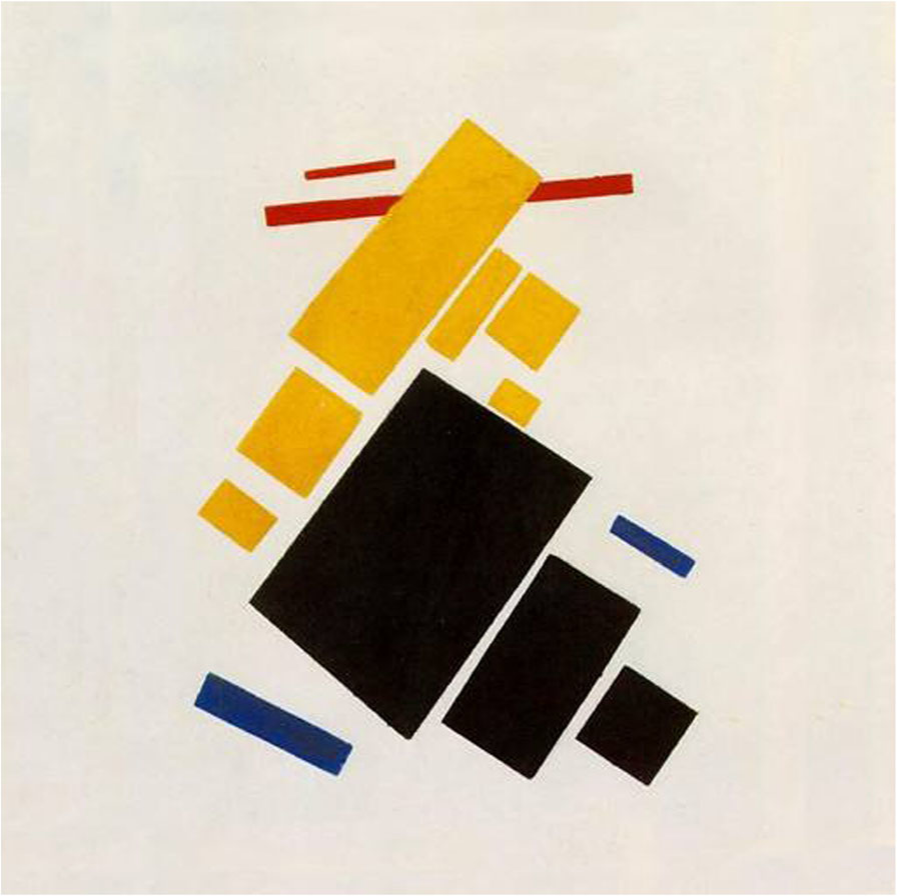
Malevich’s abstract painting example

Russian artist Kazimir Malevich is the originator of avant-garde movement, and his most famous work “Black Square” in 1913 represents the birth of supremacism. He used different types of basic supremacist elements, such as quads, ovals, crosses, triangles, circles, straight lines and semi-crescent shapes. As noted by Tao et al. [55] in Fig. 17, his works are frequently colored boldly and opaque geometric figures above a white or light colored background. In addition, a large quad determines the orientation of other subsidiary shapes.

**Fig. 18:**
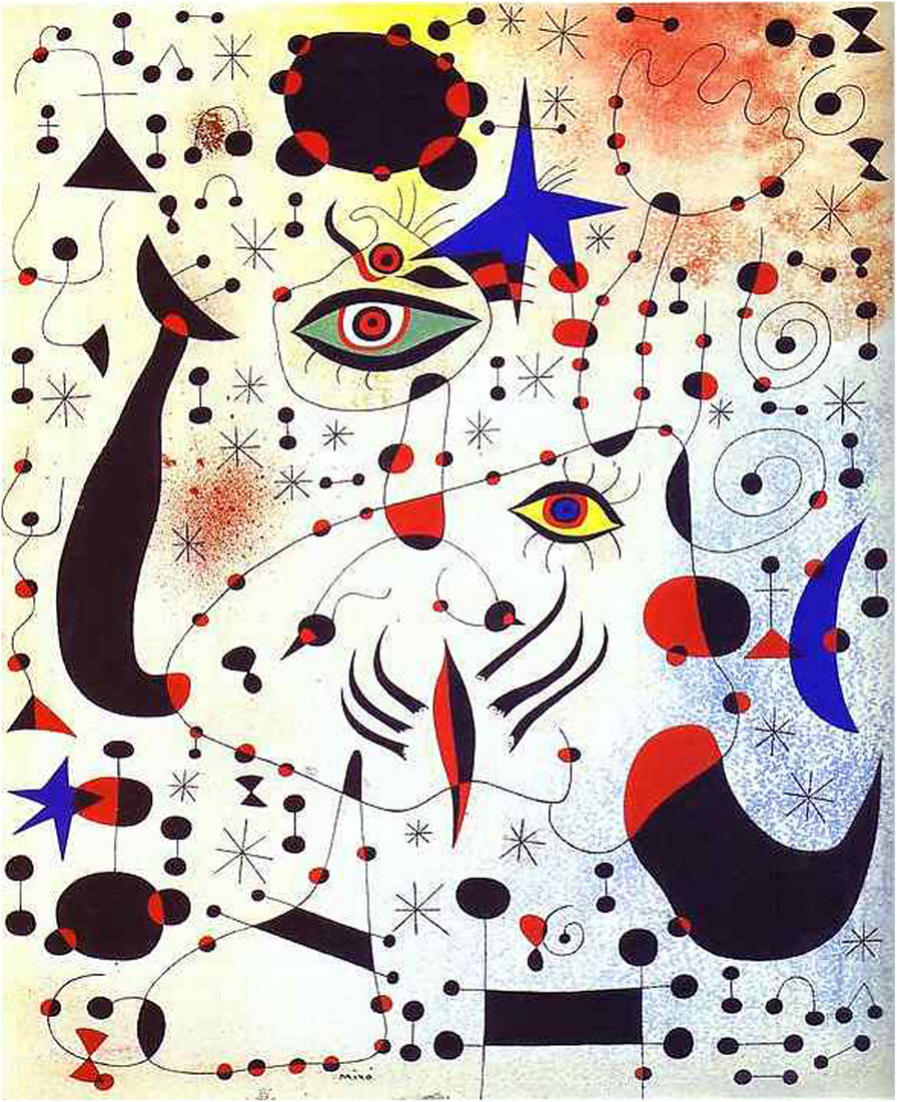
Miro’s “Ciphers and Constellations in Love with a Woman”

Prolific artist Joan Miro developed a unique style in 1920s. He arranged isolate and detailed elements in deliberate compositions. During his middle age, his art works were known as organic abstraction, featuring deformed objects as shown in Fig. 18. Xiong and Zhang [56] call these *abstract pictorial elements* according to their shapes and appearances. It is easy to find that the colors of Miro’s works are always trenchant and bright. He enjoys using a few specific colors, such as red, yellow, blue, black, and white.

**Fig. 19:**
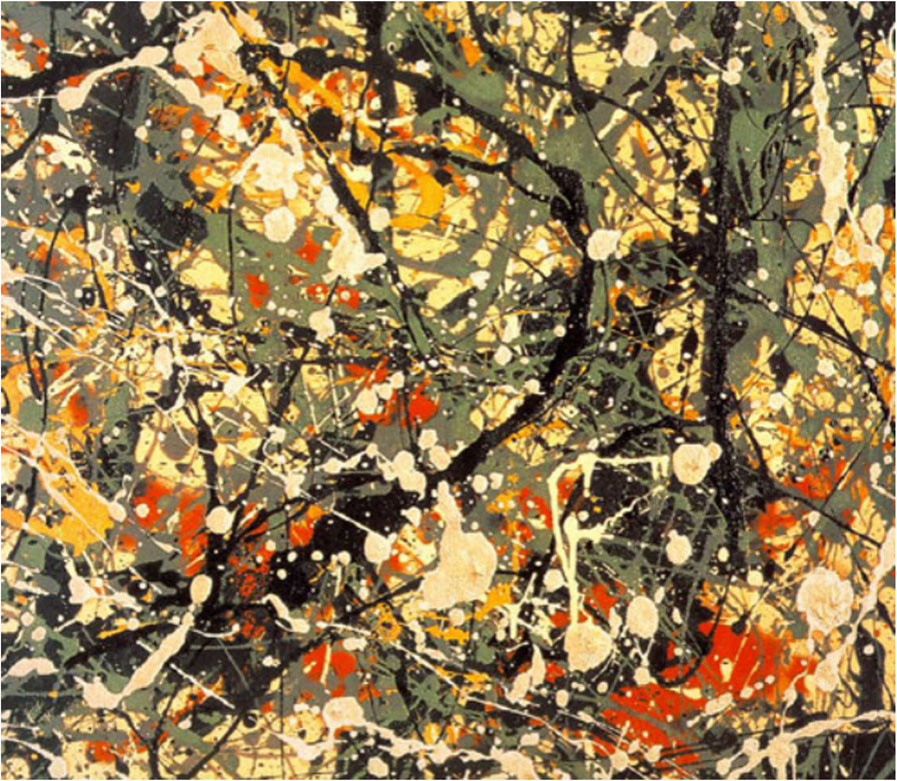
A computer generated image of Pollock’s “Number 8” [57]

Zheng et al. [57] attempt to analyze Jackson Pollock’s style, who is an influential modern American painter. He draws his paintings by dripping and pouring on canvases instead of traditional painting methods, as shown in Fig. 19. His approach is considered revolutionary for creating aesthetics, as analyzed by Taylor et al. [58] for its visual forms characterized by fractals [5]. Carefully analyzed Pollock’s various paintings, Zheng et al. [57] divide Pollock’s dripping style into four independent layers, i.e. background layer, irregular shape layer, line layer and paint drop layer from bottom up. The elements on each layer are positioned randomly.

#### Rule-based modeling

After analyzing the styles of various types of abstract paintings, researchers use different approaches to generate abstract images that mimic the original artists’ styles. The components of an abstract painting are usually interrelated. In fact, their spatial arrangements on canvas follow certain rules. For instance, in “Composition VIII” by Kandinsky, full circles with contrasting colors are often surrounded by shades with gradual changing colors and grid forms are always filled with interleaving colors.

Rule-based approach usually follows five steps:

- Step 1: Choose a specific style for automatic generation of the styled images;
- Step 2: Generate the background;
- Step 3: Decide the composition and prepare basic components;
- Step 4: Position the components based on the designed composition following the analytical rules;
- Step 5: Add texture and decoration, such as worn signs or pepper noise, if necessary.

Zhang and Yu [59] select four abstract paintings of Kandinsky from his Bauhaus period, including “Composition VIII”, “Black and Violet”, “On White II”, and “Several Circles”, to generate the artist’s style of images automatically. Based on their analysis of the paintings and reading of Kandinsky’s abstract art theories, they summarize a set of rules, for example, thin vertical and horizontal lines build the foundations and intersected by angled lines; dark boundaries are filled with light color; red and black always occur together to create a salient effect.

**Fig. 20:**
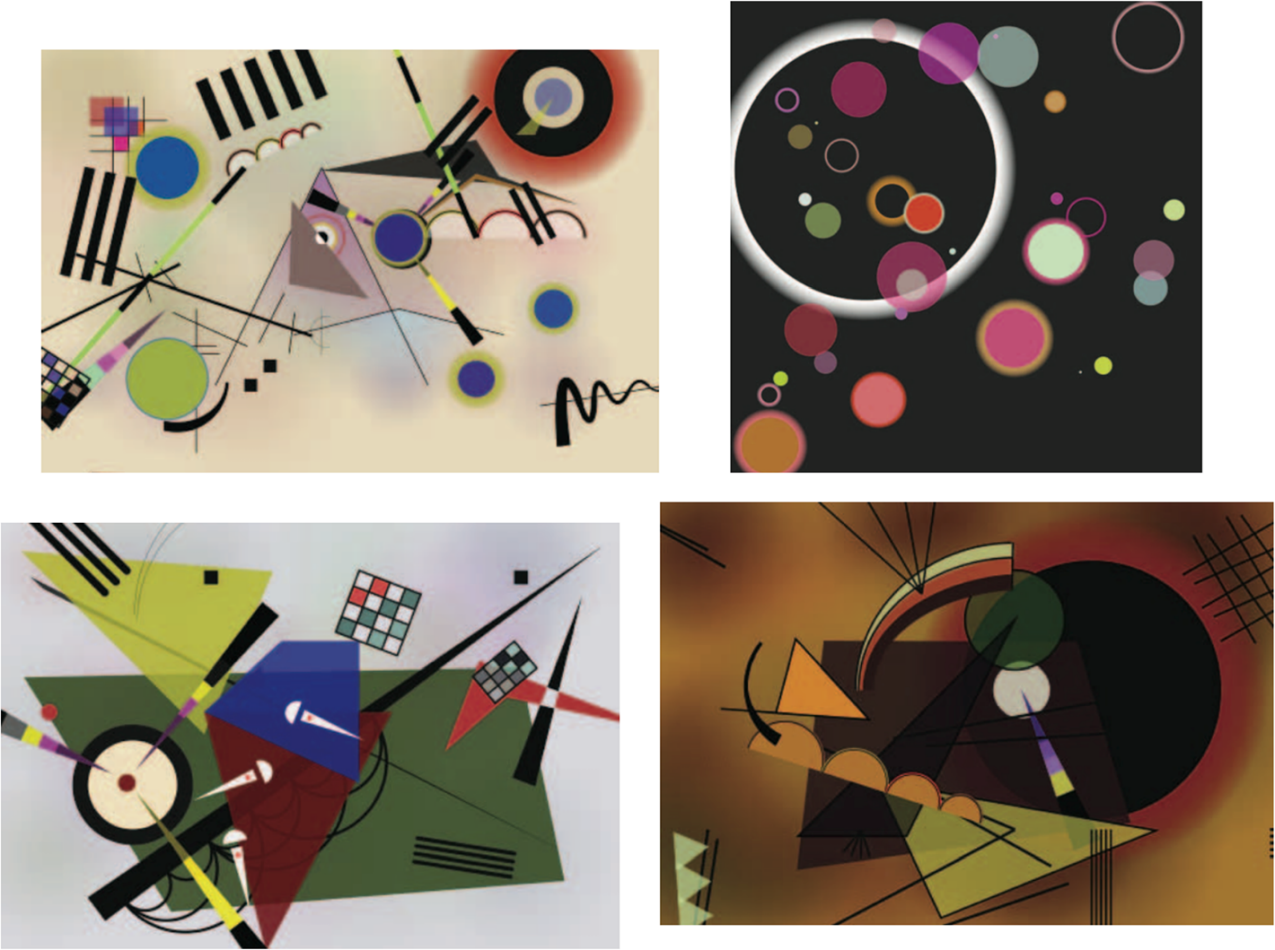
Kandinsky’s styles automatically generated: “Composition VIII” (top left), “Several Circles” (top right), “On White II” (bottom left), “Black and Violet” (bottom right) [59]

Zhang and Yu [59] parameterize various attributes of the artist’s typical components, such as boundary color, fill color, size, and location. They then use the above analytical rules to color and position the components, while randomizing other attributes. Example abstract images automatically generated using this approach are shown in Fig. 20.

**Fig. 21:**
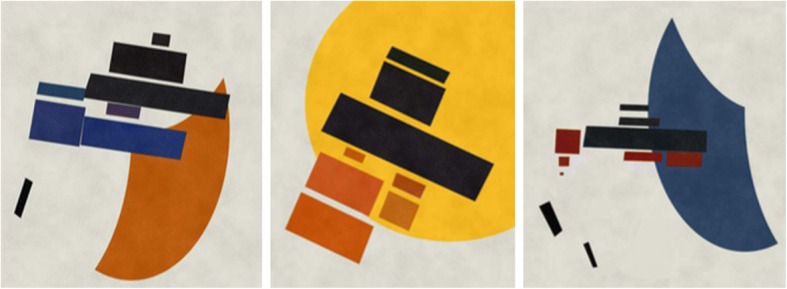
Computer generated Malevich’s *“*Mixed Shapes Style*”* [55]

Tao et al. [55] attempt to automatically generate Malevich style of abstract paintings. They first decide the color and decorations of the background, then prepare the basic components with complexities and flexibilities. Different from the generation approach of Zhang and Yu for Kandinsky style [59], they define a bounding box for each component to avoid overlaps among components, and evenly distribute components on canvas. Figure 21 gives three computer-generated results for “Mixed Shape Style”.

#### Layered approach

With non-geometrical styles, one could observe the artist’s painting process and follow the process with layers of structures and components.

**Fig. 22:**
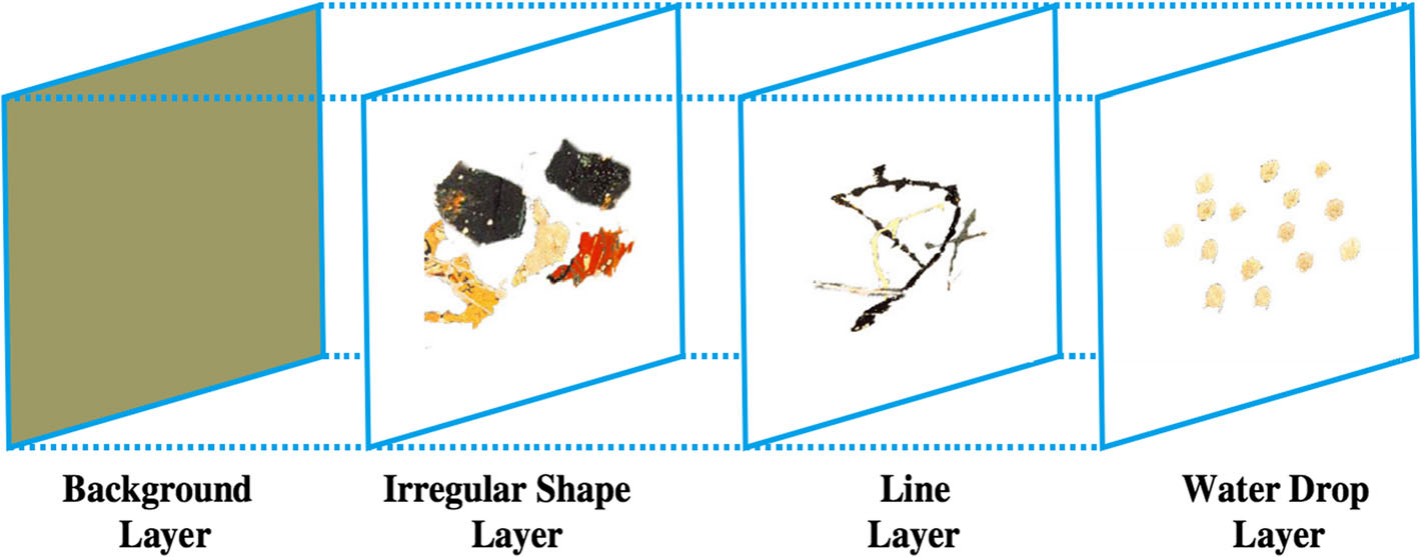
Layered approach for modeling Pollock’s drip style [57]

A typical example is Pollock’s drip style that is quite different from those of Kandinsky and Malevich. It is difficult or even impossible to come up with rules or observe regular patterns. Based on careful analysis, Zheng et al. [57] divide the structures of Pollock’s drip paintings into four independent layers, including background layer, irregular layer, line layer and paint drop layer from bottom up as shown in Fig. 22. The background layer covers the entire canvas and sets the fundamental tone of each painting. The irregular shape layer includes ellipses and polygons of random sizes. The line layer is composed of curve lines of varied lengths and widths. The top layer has all the paint drops of varied sizes. Paint drops are filled in different colors and randomly positioned on canvas. The generation order is bottom up as illustrated in Fig. 22.

Also using a layered approach, Xiong and Zhang [56] propose a process modeling approach to generating Miro’s style of abstract paintings, in the following steps:

- Step 1: Structured drawing;
- Step 2: Adaptive coloring;
- Step 3: Space filling;
- Step 4: Noise injection.

**Fig. 23:**
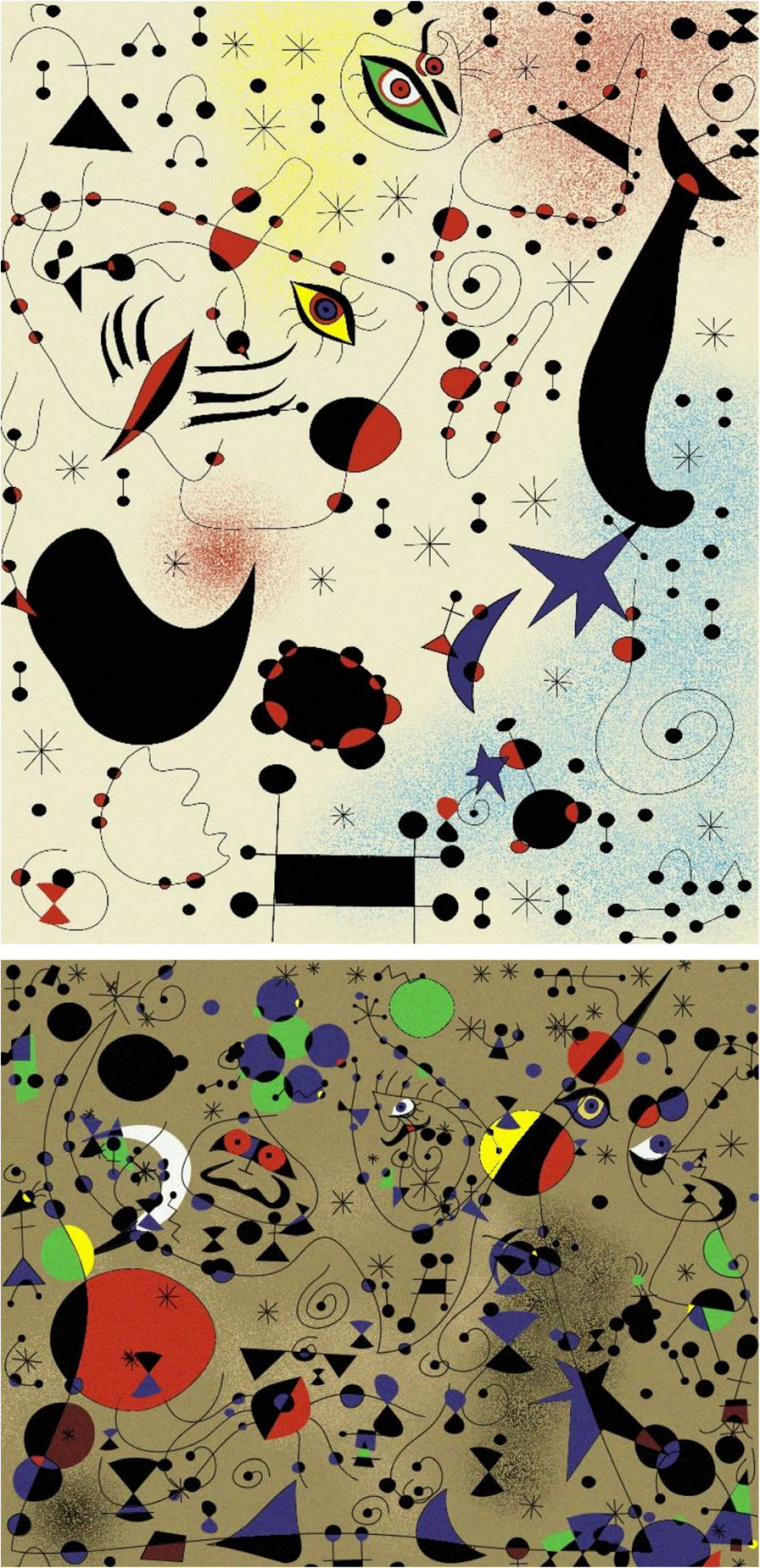
Generated images of Miro’s “Ciphers and Constellations in Love with a Woman” (top) and “Poetess” (bottom) [56]

Figure 23 shows an example of computer modeled image of “Ciphers and Constellations in Love with a Woman” and an example of generated “Poetess”, both of Miro’s well-known “Constellation” series. Of course, one could obtain varied and restructured versions of the same style by resetting or randomizing different parameters and attributes.

In summary, using the aforementioned generative methods, it is entirely feasibly that more diversified, personalized and innovative images could be generated as desired.

### Neural nets approaches

**Fig. 24:**
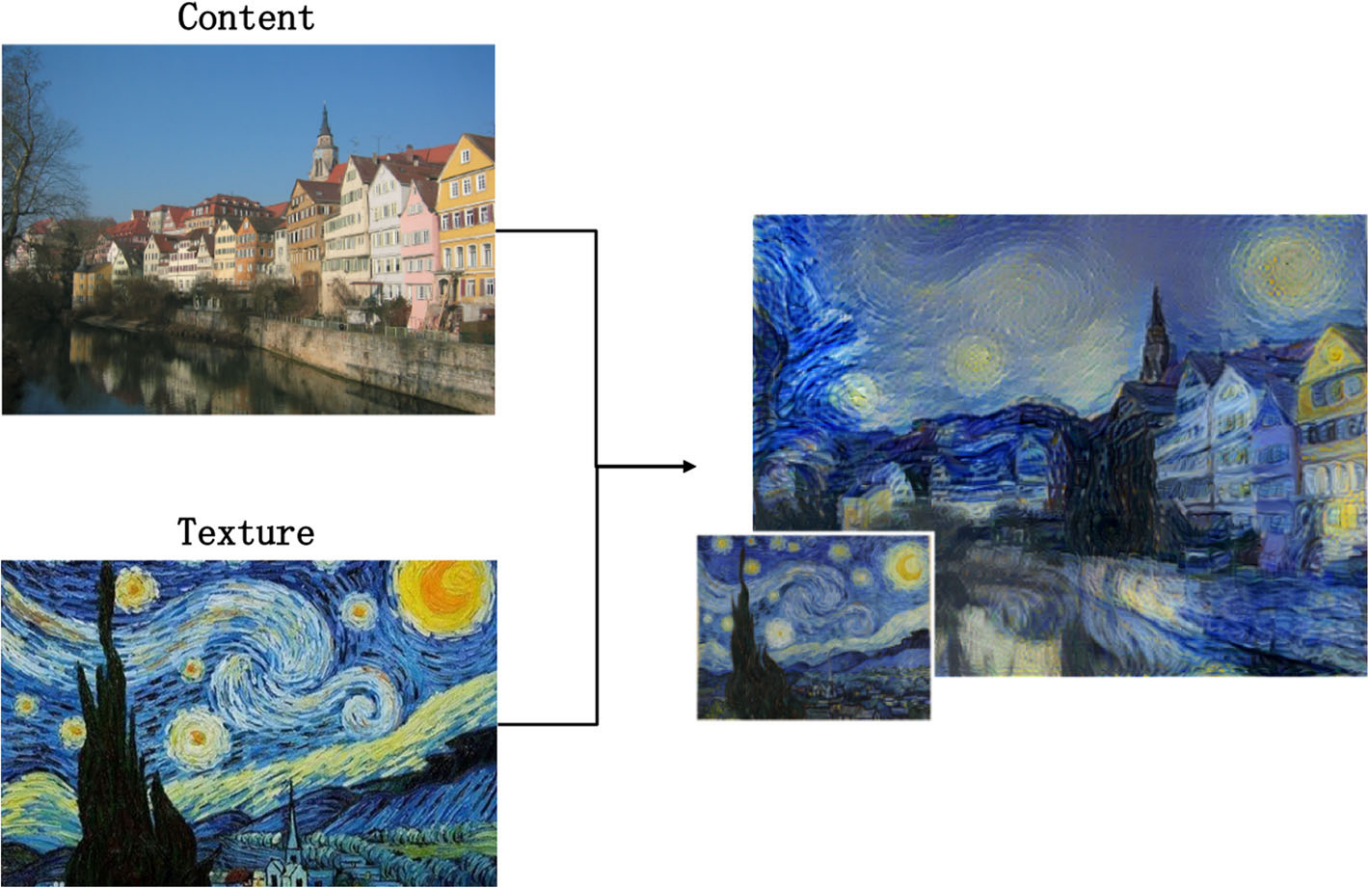
Example that combines the content of a photo with a well-known artwork

To simulate human aesthetics in depth, Gatys et al. [60, 61] proposed an image transformation approach, using a Deep Neural Network (DNN) approach. Briefly, DNN is a network constructed by layers of many small computational units. In each layer, the units are considered image filters which extract certain features from the input image. A DNN processes the visual information in a feed-forward manner hierarchically. Then, the output of the network is a feature map. Such an approach captures the texture information and obtains a multi-scale representation of the input image. Figure 24 shows an example that combines the content of a photo of Andreas Praefcke in the style of painting “The Starry Night” by Vincent van Gogh (1889).

## Discussion

### Computational aesthetics for design generation

**Fig. 25:**
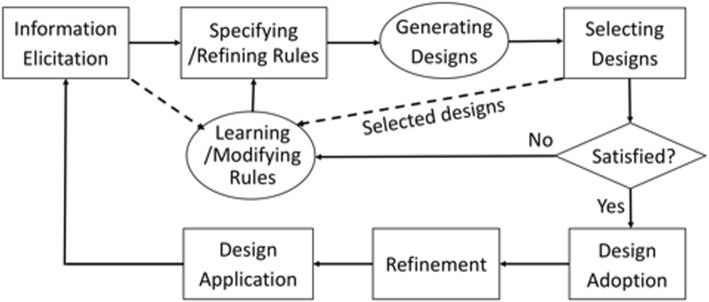
Design generation methodology

Utilizing techniques of aesthetic measurements and generative art discussed above, we propose an automatic or semi-automatic design generation framework, initially presented as a poster at VINCI’2017 [62]. In Fig. 25, rectangular boxes are manual operations and oval and diamond boxes are automatic or semi-automatic.

#### Information elicitation

Design information and requirements are collected, including sample design images partially meeting the requirements.

#### Rule specification and refinement

Based on the collected information and requirements, designers use their knowledge and experience to specify design rules, such as the logical and spatial relationships among objects, in the first round of the design process. The rules may be specified in an established formalism, such as Shape Grammar [63].

During subsequent rounds of the design, given a selected subset of all the generated designs, rules are automatically adjusted via machine learning approaches. Designers could then refine the adjusted rules.

#### Design generation

Given the set of design rules, and/or supervised learning based on the sample designs, the design generation system (e.g. shape grammar interpreter [64, 65])) automatically generates a large number of designs, while applying a set of aesthetic rules and guidelines pre-coded in the system. Deep learning algorithms, e.g., Convolutional Neural Network (CNN), can extract styles from design samples [61], such as distortion, texture, and rendering. The design rules could be responsible in generating a variety of basic designs and deep learning methods help enrich the design with the extracted design styles. In this way, each design both satisfies the design principles and has the distinct artistic style. Moreover, the framework also considers the designer’s preferences that could be modeled by personalized recommendation methods, e.g., collaborative filtering and content-based filtering. This step gives priority to the styles preferred by the designer. Some of the generated designs may not have been thought of or imagined by designers. This saves much of designers’ time, and enhances their creativity and imagination.

#### Design selection

This step is the same as in the traditional design process, except that the design choices presented are automatically generated. In digital forms, they are easily modifiable, selectable and printable.

#### Rules learning and modification

When a designer discards many designs, he/she must have used unwritten guidelines and constraints. The design generation system is equipped with an AI tool, such as a constraint solver, that can extract the constraints used by the designers, or deep learning techniques that can learn from elicited sample designs (dashed arrows). The rules involve fundamental visual elements. Deep learning methods, e.g., CNN and Deep Belief Network (DBN), could detect shapes or contours from the design samples. Based on the extracted objects, this step could formulate new elements. Deep learning methods, e.g., Sparse AutoEncoder, could learn color features from the samples to modify the existing coloring rules or generate new rules. According to the new elements, more concrete rules could be automatically learnt. These automatically-modified design rules may or may not be further refined before another round of automatic design generation.

#### Satisfied?

It may be undesirable to judge whether a design is satisfactory solely by the designer’s own subjective assessment. The selected designs are inspected by the designer and quantitatively measured for their aesthetics possibly via their complexity [45] in a semi-automatic fashion.

Quantitative aesthetic measurement methods usually include two steps: aesthetic feature evaluation and decision. In the aesthetic feature evaluation, we select the features, which represent the work best for the specific design application. One example is to objectively evaluate color and shape convexity in logo aesthetic measurement [10]. For advertisement designs, one also needs to consider saliency [9] and composition in the aesthetic measurement. In the decision step, there are two types of methods: binary classification and rating. We obtain positive or negative results by a binary classification method or a soft result providing a score ranking, which can facilitate the designer in objectively selecting designs.

By combining the judgements of the human designer and an automated approach, the design generation system could deliver a result improved over the previous round.

If one or more designs meet the requirements, they are adopted, before further **refinement** and final **design application**. This final adaption and application process would feedback to the first step, i.e. information elicitation, to help enhance the generation system. This iterative process could continue as many times as necessary until a satisfactory design is generated.

## Conclusions

This paper has introduced the current state-of-the-art of computational aesthetics. It includes two main parts: aesthetic measurement and generative art. Researchers attempt to automate the assessment of aesthetics using different features in an image. Numerous measurement approaches have been used on paintings, photographs, Chinese handwritings, webpages, and logo designs. They are also applicable to film snapshots, advertisements and costume designs in the same principle.

Generative art includes fractal art and abstract paintings generated from existing art styles, both of which can generate distinctive art works although they use totally different methods. Fractal art transforms mathematic formula into visual elements and abstract image generation models on the basic elements of the existing abstract painting styles.

Given the techniques in aesthetic measurements and generative art, one may generate aesthetic designs automatically or semi-automatically, as presented in the last section. With further development of artificial intelligence and machine learning, computational aesthetics will become easily accessible and significantly influence and change our daily life. A realistic application example would be automatic design generation as discussed above.
